# Quality of care in European home care programs using the second generation interRAI Home Care Quality Indicators (HCQIs)

**DOI:** 10.1186/s12877-015-0146-5

**Published:** 2015-11-14

**Authors:** Andrea D. Foebel, Hein P. van Hout, Henriëtte G. van der Roest, Eva Topinkova, Vjenka Garms-Homolova, Dinnus Frijters, Harriet Finne-Soveri, Pálmi V. Jónsson, John P. Hirdes, Roberto Bernabei, Graziano Onder

**Affiliations:** Department of Medical Epidemiology and Biostatistics, Karolinska Institute, Stockholm, Sweden; Department of Geriatrics, Neuroscience and Orthopedics, Catholic University, Rome, Italy; Department of General Practice and Elderly Care Medicine, EMGO Institute for Health and Care Research, VU University Medical Center, Amsterdam, The Netherlands; Department of Geriatrics, First Faculty of Medicine, Charles University, Prague, Czech Republic; HTW Berlin, University of Applied Sciences in Technology and Economics, Berlin, Germany; National Institute for Health and Welfare, Helsinki, Finland; Department of Geriatrics, Faculty of Medicine, University of Iceland, Reykjavik, Iceland; School of Public Health and Health Systems, University of Waterloo, Waterloo, ON Canada

**Keywords:** Home care, InterRAI, Assessment, Quality indicators, Performance measurement

## Abstract

**Background:**

Evaluating the quality of care provided to older individuals is a key step to ensure that needs are being met and to target interventions to improve care. To this aim, interRAI’s second-generation home care quality indicators (HCQIs) were developed in 2013. This study assesses the quality of home care services in six European countries using these HCQIs as well as the two derived summary scales.

**Methods:**

Data for this study were derived from the Aged in Home Care (AdHOC) study - a cohort study that examined different models of community care in European countries. The current study selected a sub-sample of the AdHOC cohort from six countries whose follow-up data were complete (Czech Republic, Denmark, Finland, Germany, Italy and the Netherlands). Data were collected from the interRAI Home Care instrument (RAI-HC) between 2000 and 2002. The 23 HCQIs of interest were determined according to previously established methodology, including risk adjustment. Two summary measures, the Clinical Balance Scale and Independence Quality Scale were also determined using established methodology.

**Results:**

A total of 1,354 individuals from the AdHOC study were included in these analyses. Of the 23 HCQIs that were measured, the highest proportion of individuals experienced declines in Instrumental Activities of Daily Living (IADLs) (48.4 %). Of the clinical quality indicators, mood decline was the most prevalent (30.0 %), while no flu vaccination and being alone and distressed were the most prevalent procedural and social quality indicators, respectively (33.4 and 12.8 %). Scores on the two summary scales varied by country, but were concentrated around the median mark.

**Conclusions:**

The interRAI HCQIs can be used to determine the quality of home care services in Europe and identify areas for improvement. Our results suggest functional declines may prove the most beneficial targets for interventions.

## Background

As population aging continues around the world, many older individuals express a desire to maintain independence and remain at home as long as possible. However, a substantial portion of this population will require extensive health care services in later life [1]. Adequate service provision in the home setting can have a significant impact on the quality of life of older individuals. Further, optimal care can stave off undesirable outcomes including transitions to more intensive care settings such as long-term care facilities. Understanding how well home care services meet the needs of older individuals can help evaluate the quality of care and compare service provision across jurisdictions, either within or between countries.

There are different ways of assessing the quality of care. One measure is the Outcome Assessment and Information Set (OASIS), which is used in quality measurement and care planning for home health care in the United States [2]. However, concerns about the low to moderate validity and reliability for some OASIS items, as well as concerns over its applicability in outcome measure or outcome-based quality improvement have been raised [2]. interRAI, an international research consortium specializing in the development and application of standardized assessment instruments, released its first set of home care quality indicators (HCQIs) about a decade ago [3, 4]. Second-generation HCQIs, developed in 2013, introduced several refinements to the indicators, including more sophisticated risk adjustment strategies and additional indicator domains [5]. Widespread adoption of the interRAI Home Care (RAI-HC) instrument in several North American and European jurisdictions provided very large sample sizes, which could be used to develop a more comprehensive set of risk adjusters and to introduce a two-step adjustment model involving both individual level covariates, population level stratification, and temporal adjustments [5, 6]. With a more advanced risk adjustment approach, variations in the newer HCQI scores are likely a more accurate reflection of the impact of services provided and the overall effectiveness of home care services. The ability to include data from many nations also provided better evidence of cross-national applicability than was possible with the first generation HCQIs. The advantages of interRAI’s HCQIs include more standardized items included in the assessment, a more comprehensive set of indicators and the ability to provide an aggregated measure of different HCQIs rather than a simple measure of individual HCQIs. This could be helpful in providing a more complete evaluation of the quality of care. Also, interRAI assessments are used in more than 30 countries worldwide, allowing HCQIs to be obtained from a wider geographic area.

An important contribution to interRAI’s efforts to refine the HCQIs has been the use of European data from the Aged in Home Care (ADHoC) project for both generations of indicators. The background and key findings from this work have been well described [7, 8]. The ADHoC home care data have been analyzed by Bos and colleagues [9] using the original HCQIs, confirming significant cross-country differences. However, this earlier work only included the subset of 16 prevalence HCQIs (outcome based HCQIs could not be derived based on the data available at that time) and did not have well-developed summary scales that can be generated from the second-generation HCQIs [9]. Thus, this work did not provide the comprehensive picture of quality of home care services provided in the respective countries. The purpose of this study was to apply and refine the second-generation HCQI methodology to a large European sample of individuals receiving home care and examine the quality of services. A secondary aim was to determine summary measures of services across these countries as a more comprehensive measure of home care quality.

## Methods

### Data source

Data for this study were a subset of those collected from the AdHOC Study. Methods and sample description from AdHOC have been previously published. The sample included 3785 individuals receiving home care services in 11 European countries [7]. Data were collected using the RAI-HC, a comprehensive assessment tool with more than 300 items that has been well-established as a standardized and reliable assessment instrument [10, 11]. Data were collected in each country by specially trained assessors, usually nurses, who verify information collected with sources including direct interviews of home care clients and family members, as well as review of physician reports medical records. Data were collected at baseline and at 6 and 12 month follow-ups between 2000 and 2002. Additional data about service structures and delivery were collected using a separate form and published previously [7]. The AdHOC study was funded by the Fifth Framework Programme of the European Union and ethical approval was obtained in accordance with protocols in place in all participating countries. The ethical approval from the Comitato Etico Universita Cattolica del Sacro Cuore - Policlinico A. Gemelli, Rome, Italy covered the use of anonymized data in the current study.

### Measures

#### Study sample

This study involved secondary analysis of data collected from the AdHOC study, utilizing multi-national, standardized RAI-HC data. The current study included a subset from the AdHOC sample (*n* = 3785) since necessary data to calculate HCQIs were not collected in all countries and not all individuals were followed up at 6 months. The final sample included clients from the Czech Republic, Denmark, Finland, Germany, Italy, and the Netherlands who were still living at home (*n* = 1354). In all countries except Finland, participants were invited to take part in the study and were free to decline participation. Written consent was obtained from clients of home services, their relatives, or legal guardians, with the assurance of data confidentiality. In Finland, The National Institute for Health and Welfare (formerly Stakes), holds a permission to collect data using the RAI-HC and maintain a national register based on this information. According to this permission (which is valid until 2025) no informed consent wass needed.

#### Descriptive characteristics

The full AdHOC study sample has been described previously [7], but the sub-sample included here was described using socio-demographic characteristics, geriatric conditions, disease diagnoses, and medication use using items from the RAI-HC instrument. Functional impairment was determined using interRAI’s activities of daily living (ADL) hierarchy scale, with scores derived from items on the RAI-HC [12]. Mild impairment was considered to be present in individuals with scores between 0 and 1, limited to extensive impairment was considered present in individuals with scores between 2 and 4, and those with scores of 5 or more were considered to be dependent in ADLs. Cognitive impairment was determined using the Cognitive Performance Scale (CPS), with scores of 0 to 1 representing intact cognition to borderline impairment, scores of 2 to 4 representing mild to moderate impairment and scores above 5 representing severe impairment [13]. Urinary incontinence was considered to be present if individuals were not always continent using the relevant item on the RAI-HC. Depression was determined using the Depression Rating Scale (DRS), with scores of 3 or higher being considered to represent the presence of probable depressive symptoms [14]. A binary measure for any behaviour was created using presence of any of the following: wandering, verbally or physically abusive behaviours, socially inappropriate behaviours or resisting care. The disease diagnosis section of the RAI-HC, which has been shown to collect accurate information about conditions compared to administrative databases [15], was used to determine the presence of hypertension, arthritis, dementia (including Alzheimer’s and non-Alzheimer’s types), coronary artery disease (CAD), diabetes and heart failure. The number of falls and number of medications used in the previous seven days were measured with stand-alone items on the RAI-HC.

#### Calculation of HCQIs

All 23 of the second-generation HCQIs were calculated in the current study [5]. These included eight functional indicators (decline in instrumental activities of daily living [IADLs], IADL improvement, ADL decline, ADL improvement, communication decline, communication improvement, cognitive decline, and cognitive improvement), ten clinical indicators (weight loss, injuries, falls, daily severe pain, pain not controlled, pain improvement, mood improvement, mood decline, bladder function decline, and bladder function improvement) and five social and treatment indicators (cessation of leaving home; alone and distressed; continued caregiver distress; use of hospital, emergency department [ED] or emergent care; and no influenza vaccination).

The development of these HCQIs has been described in detail elsewhere and the same methodology was used to calculate the HCQIs in this study [5]. Adjustment was an important component of the refinement of previous HCQIs and adjustment of means for each of the 23 HCQIs was carried out to account for differences in client profiles between the six countries. In this study, all covariates used in previous risk adjustment were explored and only those found to be significantly associated with the HCQIs were retained. Refer to Appendix for descriptions of each HCQI, including stratification and adjustment variables. Two summary scales were developed with the new generation HCQIs [5]. These are the interRAI Independence Quality Scale (assessing functional independence and engagement), and the interRAI Home Care Clinical Balance Quality Scale (assessing improvements in function, cognition, and psychosocial indicators). The interRAI Independence Quality Scale incorporated the following HCQIs: ADL decline, IADL decline, cognitive decline, communication decline, not going out, falls, injuries, hospitalizations/ED visits, mood decline, bladder decline, and pain not controlled. For the interRAI Home Care Clinical Balance Quality Scale, the following HCQIs were included: ADL improvement, IADL improvement, cognitive improvement, communication improvement, bladder improvement, mood improvement, pain improvement, caregiver not distressed, and not alone and distressed. Both scales range from 0 to 10 with 0 representing the worst score and 10 representing the best.

#### Statistical analyses

Descriptive statistics were calculated and HCQI development was done using SAS (version 8.2, SAS Inc., Cary NC). Risk adjustment for each HCQI was done initially using bivariate regression models and then multivariate logistic regression modelling, with the significance level set to *p* < 0.05. Summary scale development was completed using SPSS (version 18).

## Results

The general characteristics of the sample are described in Table 1. The majority of individuals receiving home care were female and between 75 and 84 years of age, with the exception of Danish participants, who were older (46.3 % over age 85). Both functional and cognitive impairment were common, with particularly high rates of both types of impairment in the German and Italian samples (16.0 and 34.2 % for ADL and 14.8 and 20.6 % for CPS, respectively). Incontinence was present in more than 40 % of individuals from each country. The prevalence of behavioural symptoms was low, while rates of depression ranged from 7.0 % in Finland to 31.9 % in the Netherlands. Both pain and falls were common among participants. Comorbidities were common with the exception of dementia, which was present in up to 20 % of the sample. Polypharmacy was also common, with 54.1 % of the total sample using more than six medications.

**Table 1:** General characteristics of European home care clients at Baseline (*N* = 1354)

	Total	Czech Republic	Denmark	Finland	Germany	Italy	Netherlands
	1354 (100)	354 (26.1)	361 (26.7)	158 (11.7)	169 (12.5)	193 (14.2)	119 (8.8)
	N (%)	N (%)	N (%)	N (%)	N (%)	N (%)	N (%)
Demographic characteristics							
Age							
Less than 75 years	262 (19.4)	60 (17.0)	39 (10.8)	39 (24.7)	43 (25.4)	57 (29.5)	24 (20.2)
75–84 years	598 (44.2)	164 (46.3)	155 (42.9)	62 (39.2)	72 (42.6)	80 (41.5)	65 (54.6)
Over 85 years	494 (36.5)	130 (36.7)	167 (46.3)	57 (36.1)	54 (32.0)	56 (29.0)	30 (25.2)
Gender Female	1034 (76.4)	284 (80.2)	284 (78.7)	128 (81.0)	126 (74.6)	119 (61.7)	93 (78.2)
Geriatric conditions							
Functional impairment (ADL Hierarchy Scale^a^ Score)							
Mild	1039 (76.7)	308 (87.0)	334 (92.5)	147 (93.0)	88 (52.1)	52 (26.9)	110 (92.4)
Limited to extensive	205 (15.1)	37 (10.5)	23 (6.4)	9 (5.7)	54 (31.9)	75 (38.9)	7 (5.9)
Dependent	110 (8.1)	9 (2.5)	4 (1.1)	2 (1.3)	27 (16.0)	66 (34.2)	2 (1.7)
Cognitive impairment (CPS^b^ score)							
Borderline impairment	964 (71.2)	244 (68.9)	296 (82.0)	123 (77.9)	109 (64.5)	96 (49.7)	96 (80.7)
Mild to moderate impairment	313 (23.1)	108 (30.5)	57 (15.8)	34 (21.5)	35 (20.7)	57 (29.5)	22 (18.5)
Severe impairment	77 (5.7)	2 (0.6)	8 (2.2)	1 (0.6)	25 (14.8)	40 (20.6)	1 (0.8)
Incontinence	609 (45.0)	149 (42.1)	146 (40.4)	70 (44.3)	85 (50.3)	100 (51.8)	59 (49.6)
Depression	262 (19.4)	103 (29.1)	36 (10.0)	11 (7.0)	25 (14.8)	49 (25.4)	38 (31.9)
Any behaviour^c^	66 (4.9)	25 (7.1)	5 (1.4)	0	22 (13.1)	12 (6.2)	2 (1.7)
Any pain	865 (63.9)	276 (78.0)	199 (55.1)	108 (68.4)	86 (50.9)	115 (59.6)	81 (68.1)
Any falls	400 (29.5)	132 (37.3)	91 (25.2)	37 (23.4)	36 (21.3)	70 (36.3)	85 (28.6)
Disease diagnoses							
Hypertension	504 (37.2)	180 (50.9)	62 (17.2)	79 (50.0)	69 (40.8)	83 (43.0)	31 (26.1)
Arthritis	422 (31.2)	213 (60.2)	94 (26.0)	50 (31.7)	13 (7.8)	20 (10.4)	32 (26.9)
Dementia	45 (8.5)	26 (7.3)	14 (3.9)	14 (8.9)	33 (19.5)	27 (14.0)	1 (0.84)
Coronary artery disease	341 (25.2)	199 (56.2)	8 (2.2)	53 (33.5)	19 (11.2)	49 (25.4)	13 (10.9)
Diabetes	302 (22.3)	109 (30.8)	38 (10.5)	54 (34.2)	51 (30.2)	24 (12.4)	26 (21.9)
Congestive heart failure	287 (21.2)	92 (26.0)	27 (7.5)	63 (39.9)	59 (34.9)	20 (10.4)	26 (21.9)
Number of medications							
0	69 (5.1)	9 (2.6)	17 (4.7)	7 (4.4)	21 (12.4)	9 (4.7)	6 (5.1)
1 to 5	552 (40.8)	107 (30.2)	160 (44.3)	34 (21.5)	64 (37.9)	117 (60.6)	70 (58.8)
6 to 9	733 (54.1)	238 (67.2)	184 (51.0)	117 (74.1)	84 (49.7)	67 (34.7)	43 (36.1)

Abbreviations: *ADL* activities of daily living, *CPS* cognitive performance scale

^a^Score of 0–1 - mild impairment; score of 2–4 - limited to maximal impairment; score of 5 or more - dependent

^b^Score of 0–1 - borderline intact; score of 2–4 - mild to moderately severe impairment; score of 5 or more - severe/very severe impairment

^c^Includes: wandering, verbally or physically abusive behaviours, socially inappropriate behaviours or resisting care

**Fig. 1:**
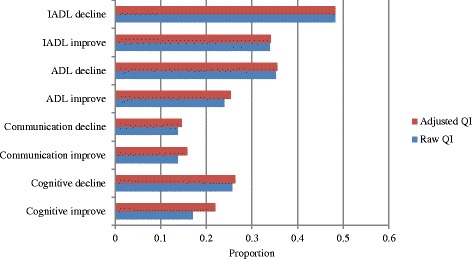
Functional Quality Indicators - average proportions of individuals declining or improving, European home care clients, *N* = 1354. Abbreviations: ADL = activities of daily living; IADL = instrumental activities of daily living; QI = quality indicator

**Fig. 2:**
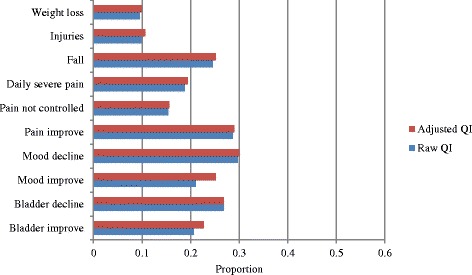
Clinical Quality Indicators – average proportions of individuals declining or improving, European home care clients, *N* = 1354. Abbreviations: QI = quality indicator

**Fig. 3:**
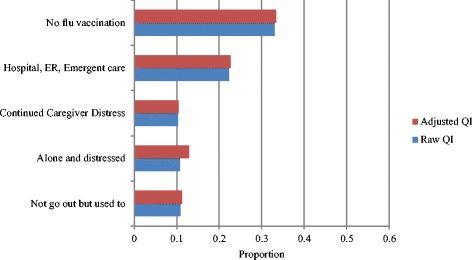
Social and Service Clinical Quality Indicators – average proportions of individuals declining or improving at 6-month follow-up, European home care clients, *N* = 1354. Abbreviations: ER = emergency room; QI = quality indicator

Figure 1 presents the raw and adjusted functional HCQIs for the sample. The highest proportion of individuals experienced decline in ADLs (48.3 %), while both improvements and declines in communication skills were least common (15.8 and 14.6 %, respectively). Among the clinical HCQIs (presented in Fig. 2), it can be seen that weight loss and injuries were less common (9.9 and 10.7 %, respectively), whereas pain improvement and mood decline were more common (29.0 and 30.0 %, respectively). From Fig. 3, no influenza vaccination was the most prevalent service QI (33.4 %), and alone and distressed was the most commonly observed social QI (12.8 %).

**Fig. 4:**
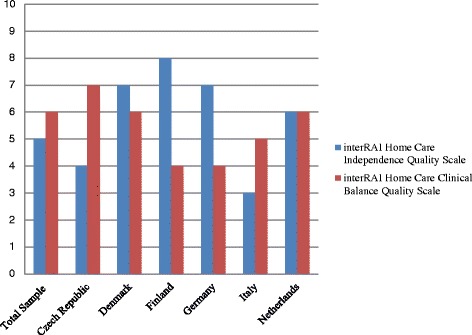
Scores by Country, interRAI Home Care Clinical Balance Scale and interRAI Home Care Independence Quality Scale, *N* = 1354

Figure 4 presents the interRAI summary scale scores by country and in the overall sample. For both the home care Clinical Balance Scale and the home care Independence Quality Scale, possible scores range from 0 (representing the worst performance) to 10 (representing the best performance). The best overall scores for the Independence Quality Scale were achieved by Finland, followed by Denmark and Germany; Italy achieved the lowest score. For the Clinical Balance Quality Scale, services in the Czech Republic performed the best, followed by those in Denmark and the Netherlands, while Finnish and German services performed the worst.

## Discussion

This study used the second-generation interRAI HCQIs to examine the quality of home care services in samples from 6 European countries. This work has shown that some indicators describe better performance of the home care service, whereas others, in particular, functional decline measures, signify areas of potentially lower quality service provision indicating areas for improvement. interRAI’s standardized data collection approach allows such comparisons of quality between countries, representing a major added value for such instruments. These instruments can be used to support care planning, intervention development and outcome measurement at the individual level and to allow performance measurement of health systems using population level data [16–18].

There was notable variance by country in the HCQIs presented in this study. The sophisticated adjustments involving many covariates to control for potentially differing client profiles in the different home care programs allowed for more accurate description of true differences in quality of home care. Noteworthy are indicators of good outcomes including IADL improvement (though there is still room for much improvement) and improvement in pain. Previous work done by the AdHOC study group found pain to be a highly prevalent problem in the overall cohort, with nearly 60 % of clients reporting it [19]. Thus, although the current results are encouraging, pain likely remains an area for continued improvement in community based individuals. More problematic outcomes were also observed, such as high rates of IADL decline. The overall rate of IADL decline observed in this study was higher than that observed in earlier work [5], though more similar rates in other functional indicators were observed. It should be noted that direct comparisons of HCQIs between countries was beyond the scope of this paper, as detailed information about policy differences between countries was not available.

The current study also builds upon earlier HCQI research that utilized ADHoC data [9]. By using the second-generation HCQIs, this study captures both prevalence and incidence based quality indicators, providing a more comprehensive understanding of the quality of home care services. Perhaps due to a more rigorous adjustment process, the estimates reported here are similar, but generally lower than those reported earlier [9]. Finally, the inclusion of two summary scales allows a high-level method of comparing between countries. The rankings generated by Bos and colleagues are similar to those of the Independence Quality Scale [9]. However, countries that performed better by this measure, such as Finland and Germany fared worse on the Clinical Balance Scale. Compared to the developmental work done previously by Morris and colleagues, this study observed similar rates of service and treatment indicators, higher rates of falls and injuries and lower rates of mood improvement [5]. The summary scale scores indicate that none of the programs in participating countries performed at the lowest level, but all have room for improvement. Such summary scales are useful in providing a high-level overview of the performance of home care programs. They can be used to provide relatively straightforward representations of complex sets of inter-related indicators in a manner that is relatively accessible to non-researchers. Policy makers, advocacy groups, managers, and the general population often wish to have one or two indicators providing a global rating of the quality of services. These two scales represent a composite of 20 separate measures of quality, providing a comprehensive but simplified representation of home care performance.

Overall this work shows that the HCQIs make it feasible to assess performance using the same items across many countries. As such, in Europe, countries can look to other examples to identify best practices and improve care. At a patient or agency level, such indicators are helpful in improving care planning, while at regional or national levels, these measures can help with benchmarking initiatives. Questions arise, however, such as how responsible a particular home care agency is for the quality of care. Such HCQIs allow the conversation about accountability in both performance and outcomes to begin. Although achieving perfect HCQI scores is unrealistic, motivating countries to improve by learning from care practices abroad is valuable.

Briefly, some limitations of the current work should be noted. First, the sample was not randomly selected, but rather was a convenience sample from AdHOC data. As the purpose of this study was to explore whether the second-generation HCQIs could be used in a European context, these data were adequate although somewhat dated. Nonetheless, it is important to note that these samples are considered to be representative of urban areas in the respective countries, but are not necessarily nationally representative. Thus, the results found here may not reflect overall quality of home care in each of the countries examined. It is also possible that some of the HCQIs are under-reported due to the study setting. For example, severe injuries may trigger transitions to long-term care and would not be captured here. Next, the HCQIs have been adjusted for a number of clinical covariates, but program characteristics and service frequency were not available. Further, it was not possible to explore potential policy differences between the countries on home care service provision, which also may have influenced the HCQIs reported here. This makes direct comparisons between countries somewhat difficult. However, all facilities were public facilities, with the exception of those in the Netherlands which were non-profit, but non-public. All facilities had public payment or compulsory insurance [7]. These similarities may mean that facility characteristics and not financial structures may explain more of the observed differences. These HCQIs have been developed in Western, developed countries and may not necessary be applicable in other countries with different policy and cultural contexts. Nonetheless, this work has demonstrated the utility of standardized assessment information from RAI-HC derived quality measures in a six country sample, demonstrating practical applications of such data to help improve quality of care. Since the AdHOC study, several new European regions (including Finland, Belgium, Italy and Ireland) have adopted the RAI-HC and these HCQIs can be useful in creating cross-national and cross-continental benchmarking for the quality of services. Finally, since the HCQIs are not routinely used and are obtained from routinely collected items within the RAI-HC itself, it is not likely that the implementation of the HCQIs would have impacted actual performance in any of the study sites. Further, it was not the primary aim of the AdHOC study to determine HCQIs, further reducing the likelihood that participation in the study altered performance.

## Conclusions

The second-generation HCQIs assess different domains of quality of home care services and are applicable in different countries. Ultimately, this work could provide a model on which to base quality measures in other care settings using standardized interRAI assessment information. These would be powerful tools in improving care delivery to vulnerable older populations in the community who are at risk of transfer to more intensive care settings. Future work could also use our results as a first standard measure of performance to which more recent performance could be compared. Matching these second-generation HCQIs to cost-effectiveness of services would also be powerful extensions of the current research and would make arguments to policy-makers more salient.

## Appendix

**Table 2:** Description of Home Care Quality Indicators including Stratification and Adjustment Covariates

HCQI	Description, stratification and adjustments
ADL improvement^a^	Clients with baseline impairment and a better score on the ADL long form.
	Stratification: IADL capacity scale score
	Adjusted for: not independent cognition, ADL decline, clinical risk, falls, hospitalizations, ADL hierarchy scale score
ADL decline^a^	Clients with a score of less than 18 on the baseline ADL long form who decline further.
	Stratification: IADL summary scale
	Adjusted for: difficulty with meal preparation, housework and bathing, unsteady gait, Cognitive Performance Scale score, institutional risk, ADL hierarchy scale score
Bladder decline^a^	Clients who experienced a decline in bladder continence (baseline score is less than 5 and lower than follow-up score). Includes clients who developed a new bladder continence problem.
	Stratification: IADL performance scale
	Adjusted for: difficulty with meal preparation, clinical risk, ADL hierarchy scale score, age over 80 years
Bladder improvement^a^	Clients who experienced an improvement in bladder continence (baseline score greater than 0 and greater than follow-up score).
	Stratification: ADL hierarchy scale score
	Adjusted for: not independent cognition, sadness, difficulty bathing, ADL decline, hospitalizations, institutional risk
Cognitive improvement^a^	Clients with some baseline cognitive impairment on the Cognitive Performance Scale who experience an improvement.
	Stratification: IADL summary scale
	Adjusted for: difficulty with phone use, impaired decision making, Alzheimer’s diagnosis, clinical risk, not independent cognition, less than 2 h of activity daily, Cognitive Performance Scale score
Cognitive decline^a^	Clients with a score of less than 6 on the Cognitive Performance Scale at baseline who experience a further decline. Includes clients who experience a new cognitive impairment.
	Stratification: IADL performance scale
	Adjusted for: difficulty with phone use, managing finances, meal preparation and bathing, falls
Communication improvement^a^	Clients with some difficulty in the communication scale (problems understanding others or making themselves understood) at baseline who experience an improvement (lower score on the communication scale).
	Stratification: IADL capacity scale
	Adjusted for: dementia (both Alzheimer’s and non), clinical risk, sadness, Cognitive Performance Scale score, ADL hierarchy scale score, age over 80 years
Communication decline^a^	Clients with a score of less than 8 on the communication scale at baseline who experience a decline (higher score on the communication scale). Includes clients with new difficulties in communication
	Stratification: IADL performance scale
	Adjusted for: difficulty managing finances, managing medications, and with phone use, Alzheimer’s disease, clinical risk, ADL hierarchy scale score
Falls	Clients who experienced one or more falls in the last 90 days.
	Stratification: clinical risk
	Adjusted for: use of assistive device, unsteady gait, ADL hierarchy scale, age over 80 years
IADL improvement^a^	Clients with a score greater than 0 on the IADL self-performance summary scale at baseline who experience an improvement (lower score).
	Stratification: clinical risk
	Adjusted for: sadness, ADL decline
IADL decline^a^	Clients with a score less than 15 on the IADL self-performance summary scale at baseline who declined (had a higher score).
	Stratification: clinical risk
	Adjusted for: difficulty with meal preparation and housework, institutional risk, ADL hierarchy scale score
Injuries^a^	Clients with new injuries - fractures, second- or third-degree burns or unexplained injuries – since baseline.
	Stratification: clinical risk
	Adjusted for: ADL decline, pain, unsteady gait
Mood improvement^a^	Clients with fewer depressive symptoms on the Depression Rating Scale at follow-up.
	Stratification: IADL summary scale
	Adjusted for: ADL decline, hospitalizations, depression rating scale score
Mood decline^a^	Clients with more depressive symptoms on the Depression Rating Scale at follow-up. Includes clients with new depressive symptoms.
	Stratification: ADL hierarchy scale
	Adjusted for: clinical risk, difficulty bathing, institutional risk
Pain not controlled	Clients who have pain and are receiving inadequate pain control or no pain medication.
	Adjusted for: clinical risk
Pain improvement^a^	A reduction in pain since baseline.
	Stratification: clinical risk
	Adjusted for: unsteady gait, Cognitive Performance Scale score
Daily severe pain^a^	Individuals with at least daily episodes of severe pain at follow-up.
	Stratification: clinical risk
	Adjusted for: dyspnea, unsteady gait, ADL long form score, ADL short form score, depression rating scale score
Continued caregiver distress	Clients with caregivers who express distress, anger and or depression at baseline and follow-up.
	Stratification: Cognitive Performance Scale score
	Adjusted for: not independent cognition, IADL difficulty, difficulty with locomotion, impaired decision making, difficulty with housework, clinical risk
Alone and distressed^a^	Clients who are distressed by a decline in social activities and are alone for long periods or all the time at follow-up.
	Stratification: clinical risk
	Adjusted for: not independent cognition, pain, unsteady gait, Cognitive Performance Scale score, ADL hierarchy scale score, depression rating scale score
Used to go out^a^	Clients who compared to the baseline assessment, go out less or not at all.
	Adjusted for: IADL difficulty, Cognitive Performance Scale score
No flu vaccine	Clients who did not receive an influenza vaccination at either baseline or 6-month follow-up assessments
	Stratification: clinical risk
	Adjusted for: less than 2 h of daily activity, institutional risk, Cognitive Performance Scale score, depression rating scale score
Hospitalization and Emergency Department use^a^	Clients who have been hospitalized or visited the emergency department in the 90 day period before the follow-up assessment.
	Stratification: IADL capacity scale score
	Adjusted for: physician visits, clinical risk, diabetes, depression rating scale score
Weight loss^a^	Clients with any unintended weight loss at follow-up.
	Adjusted for: clinical risk

Abbreviations: *ADL* activities of daily living, *IADL* instrumental activities of daily living

^a^Measured at the 6-month follow-up assessment

## Abbreviations

AdHOC: Aged in the home care
ADL: Activities of daily living
CAD: Coronary artery disease
CHF: Congestive heart failure
CPS: Cognitive performance scale
DRS: Depression rating scale
ED: Emergency Department
HCQI: Home care quality indicators
IADL: Instrumental activities of daily living
RAI-HC: InterRAI resident assessment instrument - home care
